# Geminiviral Triggers and Suppressors of Plant Antiviral Immunity

**DOI:** 10.3390/microorganisms9040775

**Published:** 2021-04-08

**Authors:** Ruan M. Teixeira, Marco Aurélio Ferreira, Gabriel A. S. Raimundo, Elizabeth P. B. Fontes

**Affiliations:** Department of Biochemistry and Molecular Biology, BIOAGRO, National Institute of Science and Technology in Plant-Pest Interactions, Universidade Federal de Viçosa, Viçosa 36571.000, MG, Brazil; ruanmaloni@gmail.com (R.M.T.); marco.aurelioferreira@hotmail.com (M.A.F.); gabriel88saraiva@gmail.com (G.A.S.R.)

**Keywords:** PAMP-triggered immunity, effector-triggered immunity, RNA silencing, viral suppressors, NIK1, PTI, ETI, geminiviruses

## Abstract

Geminiviruses are circular single-stranded DNA plant viruses encapsidated into geminate virion particles, which infect many crops and vegetables and, hence, represent significant agricultural constraints worldwide. To maintain their broad-range host spectrum and establish productive infection, the geminiviruses must circumvent a potent plant antiviral immune system, which consists of a multilayered perception system represented by RNA interference sensors and effectors, pattern recognition receptors (PRR), and resistance (R) proteins. This recognition system leads to the activation of conserved defense responses that protect plants against different co-existing viral and nonviral pathogens in nature. Furthermore, a specific antiviral cell surface receptor signaling is activated at the onset of geminivirus infection to suppress global translation. This review highlighted these layers of virus perception and host defenses and the mechanisms developed by geminiviruses to overcome the plant antiviral immunity mechanisms.

## 1. Introduction

Geminiviruses are circular single-stranded DNA viruses grouped into one of the largest and most successful families of plant viruses (*Geminiviridae* family) [1]. Collectively, the geminiviruses cause devasting diseases in a large variety of economically relevant crops and vegetables, resulting in the most diverse symptoms. The broad-range host spectrum of the viruses from the Geminiviridae family may be associated with the large capacity of geminiviruses to overcome the multilayered antiviral immune system of the plant cell, which is broadly divided into RNA interference (RNAi), pathogen-associated molecular pattern (PAMP)-triggered immunity (PTI), and effector-triggered immunity (ETI) (see Abbreviations) [2]. Signaling from the cell surface, PTI is the first line of plant defense mediated by immune pattern recognition receptors (PRRs), which detect and interact with conserved molecular motifs from the pathogens, PAMPs [3]. As a second line of defense, ETI is mediated by intracellular immune receptors that specifically recognize, directly or indirectly, viral effectors delivered into the cytosol by the pathogens.

RNAi or RNA-silencing-derived antiviral immunity is a well-characterized plant antiviral immunity mechanism, which has been shown to operate against virtually all plant viruses [4,5]. Likewise, ETI, also referred to as resistance (R) gene-mediated immunity, has long been recognized as an efficient plant defense layer against viruses [6,7]. Studies with plant viruses pioneered in describing hallmarks in ETI responses, including the hypersensitive response (HR), salicylic acid accumulation, and systemic acquired resistance (SAR) [8,9,10]. In addition, several viral effectors (avirulence gene products) and their cognate R proteins have been characterized [2]. Emerging evidence has demonstrated that the classical PTI characterized in nonviral pathogen–plant interactions also operates against plant viruses [11,12]. These multilayered immune defenses are activated and suppressed by viral components or effectors, functioning as virulence factors in susceptible genotypes and as avirulence (Avr) factors in resistant genotypes.

Like any other plant virus, geminiviruses both activate and suppress RNA-silencing-mediated antiviral immunity. Infected hosts accumulate geminivirus-derived small interfering RNA (siRNA) of 21–24 bp, and almost all geminivirus proteins have been shown to suppress critical steps in the RNA-silencing mechanism [13]. Evidence that viral PTI functions against geminiviruses includes the finding that PTI upstream receptors are virulence targets of geminivirus proteins, which can also suppress downstream PTI-like responses, fulfilling the concept that PTI must be inhibited for successful infection [14,15]. Likewise, some geminiviruses proteins have been shown to activate and suppress ETI-like responses [16]. As further evidence that plants deploy ETI to fight geminiviruses, the tomato Ty-1 locus, which confers resistance to tomato yellow leaf curl virus (TYLCV), encodes a nucleotide-binding leucine-rich repeat (NLR) domain-containing protein, a reminiscent structural configuration of typical ETI receptors [17]. This review focuses on RNA silencing and the antiviral innate immunity mechanisms that plants deploy to fight viruses and the strategy that geminiviruses evolved to overcome these defense barriers. Virtually all geminiviral proteins, which carry a primary function (movement, replication, encapsidation) required to complete the virus life cycle, evolved to accommodate virulence functions as well. Although not covered in this review, hormone signaling has been shown to be connected with anti-geminiviral immunity. For in-depth information on this topic, the reader is referred to a recent review in the molecular interplay between hormones and geminiviruses [18].

## 2. Structural and Functional Organization of the Geminivirus Genome

The *Geminiviridae* family encompasses circular single-stranded DNA viruses that are packed into icosahedral, geminate virion particles. The family is further divided into nine genera (*Begomovirus, Mastrevirus*, *Capulavirus*, *Curtovirus*, *Becurtovirus*, *Eragrovirus*, *Grablovirus*, *Topocuvirus*, and *Turncurtovirus*), according to the genome organization and phylogenetic relationship of the geminivirus species and types of the transmissible insect vectors [19].

**Figure 1:**
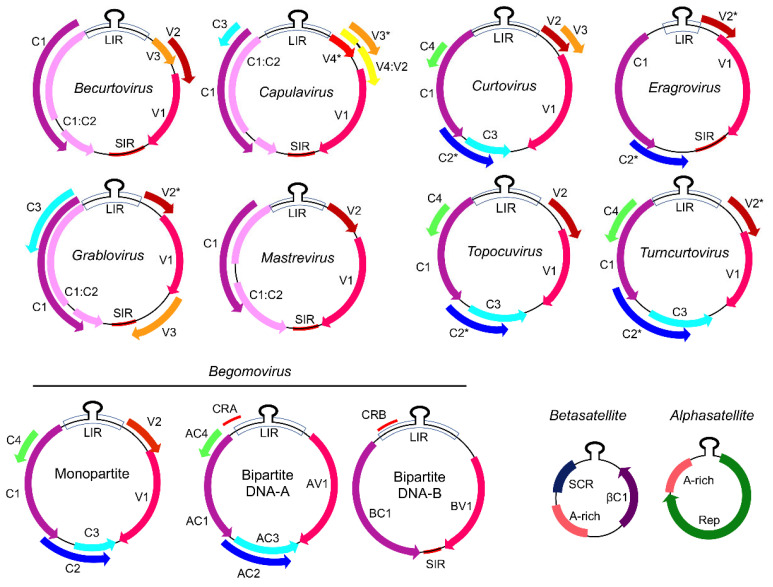
Genomic organization of geminiviruses (Geminiviridae family). The Geminiviridae family includes nine genera represented by monopartite or bipartite species. LIR denotes the long intergenic region; SIR, the short intergenic region; and CR, the common region. The open reading frame (ORF) C1/AC1 encodes the replication initiator protein (Rep) and C3/AC3 encodes the replication enhancer protein (Ren), which are associated with replication. The ORF C2/AC2 encodes a transcriptional activator protein (TrAP) that controls the transcription of viral and host genes; C4/AC4 is a virulence factor. The capsid protein (CP) is indicated in the monopartite and bipartite genomes, as V1 and AV1. In monopartite species, V2 represents the movement protein (MP). V3, present in some genomes, is an inhibitor of gene silencing. ORFs with asterisks (*) have not been functionally assayed. In bipartite begomoviruses, MP (BC1) is encoded by the DNA-B, which also encodes the nuclear shuttle protein, NSP (BV1), which facilitates the nucleocytoplasmic movement of viral DNA. Bipartite begomoviruses are often associated with ssDNA satellites, i.e., the alphasatellites, which encode a replication protein (Rep), and the betasatellites, which encode the virulence-related βC1 protein. A-rich is a conserved adenine rich region of the DNA satellites, and SCR is the satellite conserved region. Adapted from [1]

The geminiviruses can be either monopartite with a single genomic configuration (DNA-A-like) or bipartite with two genomic components, designated DNA-A and DNA-B, with a coding capacity ranging from 4 to 8 viral proteins (Figure 1). Their genome is transcribed into bidirectional transcriptional units from the origin of replication (Ori). The functional structures of Ori include a conserved stem-loop structure and the nonanucleotide sequence TAGTATTAC that constitutes the site of replication initiation by the viral replication initiator protein (Rep), encoded by the complementary-sense strand and, hence, also designated C1 (AC1) [1]. The remaining complementary-sense strand-encoded viral proteins are designated C2 (AC2), which functions as a transcriptional activator protein (TrAP); C3 (AC3), or replication enhancer protein (REn); C4 (AC4), a pathogenicity determinant; and C5 (AC5), not identified in all geminivirus genomes. The virion strand encodes V1 (AV1); the coat protein (CP); V2 (AV2), which exhibits movement functions; and V3, not present in all geminivirus genomes.

The B component of bipartite geminiviruses encodes BC1 and BV1. BV1 is a nuclear shuttling protein (NSP) that facilitates the intracellular transport of viral DNA from the nucleus to the cell periphery and assists BV1, the classic movement protein (MP), to move the viral DNA to the adjacent, uninfected cell via plasmodesmata [20]. Some geminiviruses are often associated with DNA satellites, designated beta- and alphasatellites, which affect geminiviral pathogenicity and symptom development [21,22] (Figure 1). The genus *Betasatellite* belongs to the family *Tolecusatellitidae*, whereas the *Alphasatellite* genus belongs to the *Alphasatellitidae* family. These circular ssDNA satellites are approximately 1–1.4 kb ssDNA; alphasatellites encode a replication-associated protein (Rep), whereas the betasatellites encode βC1 involved in symptom induction and suppression of transcriptional and post-transcriptional gene silencing.

## 3. RNA-Silencing-Mediated Antiviral Mechanisms

RNA silencing, also designated RNA interference (RNAi), is a primary antiviral defense mechanism of plant cells that is activated by double-stranded (ds) RNAs [4,23]. The dsRNA-induced gene-silencing mechanisms are divided into three phases: biogenesis, amplification, and effector phases. The type III RNases dicers (DCLs) carry out the siRNA biogenesis phase by recognizing and processing dsRNA derived from several sources, including viral dsRNA or micro (mi)RNA precursors. DCL2 and DLC4 generate short dsRNAs of 21 and 22 nucleotides (nt), whereas DCL3 processes dsRNA precursors into 24-nt siRNAs [24,25,26,27,28].

**Figure 2:**
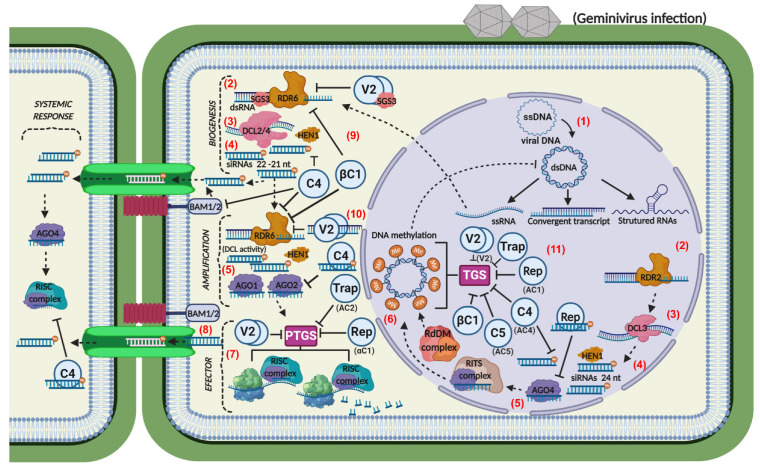
Geminivirus-induced RNA silencing and viral suppressors. Geminivirus particles are delivered into the cytoplasm, where they are uncoated and CP-bound viral ssDNA complexes are imported into the nucleus. (1) In the nucleus, the viral ssDNA is converted into dsDNA, the replicative form. (2) Then, viral ssRNAs are synthesized and are recognized by the RNA-silencing machinery, initiating the biogenesis phase by converting ssRNA to dsRNA via RDR2 (RNA-dependent RNA polymerase 2 in the nucleus) or RDR6 (in the cytoplasm) with the help of suppressor gene silencing 3 (SGS3), which stabilizes the substrates for (3) dicer (DCL)-mediated processing into 21-, 22-, or 24-nt siRNAs (virus-derived small interfering RNA; vsiRNA). (4) HUA Enhancer 1 (HEN1)-methylated siRNAs are then amplified by RDR1 or RDR6 enhancing siRNA-mediated viral immunity. (5) During the effector phase, Argonaute 4 (AGO4) and AGO1/2 (in the cytoplasm) interact with siRNAs to form RNA-induced transcriptional silencing complex (RITS) and RNA-induced silencing complex (RISC), respectively. (6) In the nucleus, RITS targets the viral transcribed genome and sequesters the RNA-dependent DNA methylation (RdDM) complex, which remodulates the chromatin for transcriptional gene silencing (TGS) of the viral genome. (7) In the cytoplasm, RISC mediates post-transcriptional gene silencing (PTGS) inhibiting the transcription of viral genes via degradation of viral mRNAs. (8) Viral siRNAs generated in the biogenesis and amplification phases are systemically spread via plasmodesma in a barely any meristem 1 (BAM1)- or BAM2-dependent process. For successful infection, geminiviral suppressors of RNA silencing or (VSRs) can act at all levels of TGS and PTGS to suppress siRNA- or RNA interference-mediated antiviral immunity. (9) AC4/C4, Rep, βC1, and V2 inhibit biogenesis of siRNA; (10) βC1, V2, AC2/C2, C4 suppress amplification of siRNA; and (11) βC1, Rep, AC2/C2, and V2 impair the effector phase. Furthermore, the geminivirus suppressors of RNA silencing, Rep, V2, AC4/C4, AC2/C2, and AC5, may interfere with downstream events of TGS. AC4 also interferes with siRNA systemic translocation by targeting BAM1/2. Some geminiviral suppressors interfere with RNA silencing by targeting siRNA (Rep and C4) and long non-coding RNAs (lncRNAs; V2). They also act by activating or inducing the expression of endogenous suppressors of RNA silencing. See Figure 1 for the designations of the viral proteins. The figure was created with BioRender.com.

As a plant DNA virus, geminiviruses can generate dsRNA triggers (precursors) via different mechanisms, including overlapping transcripts from a divergent transcription of virus genes, structured (hairpin) transcripts, or dsRNA synthesized from viral mRNA by the host RNA-directed RNA polymerase (RdRP or RDR) (Figure 2). These dsRNA precursors are then cleaved by DCLs and converted into virus-derived small interfering RNAs (vsiRNAs) [29,30]. In the amplification phase, vsiRNAs are amplified by RdRP or RDR and are subsequently stabilized by HUA enhancer 1 (HEN1)-mediated 2′O-methylation, which protects the 3’-end of siRNAs from uridylation activity and subsequent degradation [31,32,33]. 

The effector phase initiates with the assembly of newly synthesized vsiRNAs with one member of the effector AGO (argonaute) family into RNA-induced silencing complexes (RISCs) or RNA-induced transcriptional silencing complexes (RITSs), which target complementary RNA or DNA for silencing (Figure 2) [34,35,36]. RISC acts at the post-transcriptional level and targets viral RNAs for degradation through cleavage (slicing) or translational arrest, leading to post-transcriptional gene silencing (PTGS). RITS is involved in transcriptional gene silencing (TGS) through DNA or histone methylation and heterochromatin formation.

In PTGS-mediated antiviral defense, DCL2 and DCL4 often process dsRNA precursors into 21- and 22-nt siRNAs that interact with AGO1 and AGO2 [28,30,37]. PTGS predominantly involves RNA cleavage via the endonucleolytic activity of AGO1 [38]. Recent studies have identified AGO-mediated translational repression as an additional RNA-silencing mechanism against plant viruses [39,40,41,42]. Loss-of-function mutations have implicated AGO1 and AGO10 in translational repression, whereas the AGO2-mediated translational suppression has been studied through an in vivo reporter assay [43,44,45,46]. The mechanism of AGO-mediated translational repression in plants is still poorly understood, but it seems to depend on the complementary site for siRNA or miRNA on mRNA. Targeting the 5′UTR enables AGO1-RISC to sterically hinder ribosomal recruitment [45], whereas siRNA targeting sites within the open reading frame (ORF) may impair ribosome elongation [45]. At the 3′ UTR on mRNA, AGO1-RISC may repress translational initiation by interfering with 48S initiation complex formation, resembling the mechanism observed in animal cells [45,47].

Plants also deploy siRNA-directed TGS as an antiviral defense against DNA viruses [48]. The TGS mechanism is often used by plant cells to control endogenous gene expression. In this case, the RNA polymerase (pol) IV transcript is converted into dsRNA by RNA pol IV-associated RDR2 (RNA-dependent RNA polymerase 2) [49]. Then, DCL3 cleaves dsRNA to produce 24-nt siRNA, which is transported to the cytoplasm for AGO4 loading and RITS assembling. The AGO4:siRNA complex is redirected to the nucleus to target the complementary transcript of RNA polymerase V, synthesized in the opposite direction from the RNA pol IV transcript. Therefore, siRNA correctly positions AGO4 that recruits the Novo DNA methyltransferase DRM2-containing RNA-directed DNA methylation (RdDM) complex to methylate DNA on the target locus [49,50,51,52]. The RNA-directed DNA methylation is often directed to promoter regions to interfere with gene expression at the transcriptional level.

## 4. Geminiviral Suppressors of PTGS and TGS

As an adaptive antiviral defense mechanism, RNA silencing is induced during infection, and virus-derived siRNAs accumulate at high levels. Likewise, geminivirus infection induces siRNA accumulation [24]. Cabbage leaf curl virus (CabLCV) infection in *Arabidopsis* and African cassava mosaic virus (ACMV) infection in *Nicotiana benthamiana* and cassava have been associated with the biogenesis of 21-, 22-, and 24-nt vsiRNAs derived from the coding and the intergenic regions of these geminiviruses [5,24,53,54]. In these previous studies, all 24-nt vsiRNAs and a large fraction of 22-nt vsiRNAs were generated by DCL3 and DCL2, respectively, demonstrating the assembly of several distinct silencing pathways in geminivirus–plant interactions [24]. In addition to siRNAs, long non-coding RNAs (lncRNAs) accumulate in plants infected by TYLCV [55]. These long RNAs can be derived from intergenic sequences and antisense sequences of natural transcripts and may mimic endogenous targets to compete with TYLCV-related siRNAs by a yet unknown mechanism.

Antiviral TGS is considered a primary defense pathway against geminiviruses. Accordingly, reverse genetics studies have demonstrated that loss-of-function mutants of TGS components increase hypersensitivity to geminivirus infection and interfere with host recovery [48,52]. DCL3 is essential to combat DNA viruses because antiviral immunity persists in *dcl2* and *dcl4* mutants but not in *dcl3* mutants. Loss of *DCL3* function enhances *Begomovirus* pathogenicity and abolishes 24-nt vsiRNAs biogenesis [24,52]. In TGS, geminiviral ssDNA is converted into the replicative form dsDNA, which is recognized by RNA polymerase IV, in the nuclei of infected cells [48]. The RNA pol IV-transcribed single-stranded RNA is converted to dsRNA by RDR2 and then processed by DCL3 into 24-nt vsiRNAs. AGO4 is loaded with 24-nt vsiRNAs, which target the complementary nascent transcript of the RNA pol IVb complex, recruiting the RdDM complex for viral DNA methylation. The Histone H3 lysine 9 (H3K9) methyltransferase (kyp2/suvh4) is also involved in chromatin modification of the viral genome, resulting in epigenetic modification of the viral genome [56].

Many plant viruses have evolved viral suppressors of RNA silencing (VSRs) as a virulence strategy [57,58,59,60]. The geminiviral VSRs are multifunctional proteins displaying host defense-suppressing activities and viral life cycle–supporting functions. They can interfere with both PTGS and TGS in all three steps of the processes (Figure 2). They also act downstream of TGS, directly or indirectly affecting DNA methylation [13]. Mechanistically, geminiviral VSRs either suppress the activity or repress the accumulation (expression) of RNA-silencing machinery components.

Rep, also designated AC1 (bipartite geminiviruses) and C1 (monopartite geminiviruses), has been shown to function as an efficient VSR at both PTGS and TGS (Figure 2). Tomato yellow leaf curl Sardinia virus (TYLCSV) Rep directly reduces methyltransferase *1* (*MET1*) and chromomethylase 3 (*CTM3*) expression in infected plants, interfering with the cycle of DNA methylation and TGS (Figure 2) [61]. Wheat dwarf virus (WDV) Rep suppresses PTGS by binding to 21-nt single-stranded and double-stranded vsiRNAs, thereby preventing their association with the respective AGOs to impair RNA silencing of viral genes [62]. Likewise, TrAP (AC2 or C2) suppresses antiviral TGS and PTGS via different mechanisms depending on the cognate TrAP-encoding geminivirus genome. Among the effective mechanisms for suppressing RNA silencing, TrAP either directly interacts with silencing host factors or transactivates expression of host suppressors of RNA silencing; thereby, the TrAP silencing–suppressing function is often coupled to its transcriptional activity that targets and transactivates not only viral gene promoters but also host genes [63,64,65,66]. Both AC2 and C2 have been shown to interact with and inhibit common host RNA-silencing factors, including adenosine kinases (ADKs), H3K9me2 histone methyltransferase, SU(VAR)3-9 homolog 4/kryptonite (SUVH4/KYP) involved in TGS [66,67,68], AGO1 and RDR6, involved in PTGS [69], and calmodulin-like protein (rgs-CaM), an endogenous suppressor of PTGS [63,65]. Beet severe curly top virus (BSCTV) C2 also interacts with and stabilizes S-adenosyl-methionine decarboxylase 1 (SAMDC1); thereby, C2 interferes with the host defense mechanism of DNA methylation-mediated gene silencing by attenuating the 26S proteasome-mediated degradation of SAMDC1 [70]. In addition to directly interacting with calmodulin-like protein 39 (rgsCaM), AC2 from tomato golden mosaic virus (TGMV) induces the expression of *rgsCaM*, which may target silencing suppressors of RNA viruses for degradation via the autophagy pathway [63]. Likewise, AC2 proteins from the mungbean yellow mosaic virus-*Vigna* (MYMV) and ACMV have been shown to target promoters and regulate gene expression of endogenous silencing suppressors, including the Werner exonuclease-like 1 (*WEL1*) gene [65].

C4/AC4 is also part of the virus arsenal against antiviral RNA silencing by interacting and sequestering dsRNA precursors from DCL cleavage and siRNA from RISC loading [71,72]. C4 interacts with 21-nt vsiRNAs to prevent the spread of vsiRNAs and, hence, suppresses RNA silencing systemic immunity [72,73]. TYLCV C4 strongly associates with the intracellular kinase domain of the plasmodesma-localized RLKs, barely any meristem 1 (BAM1) and BAM2, required for systemic immunity [73]. C4 from cotton leaf curl Multan virus (CLCuMuV) also interacts with and inhibits *S*-adenosyl methionine (SAM), the universal donor of methyl groups in methylation reactions [74]. Therefore, C4 decreases SAM and HEN1 activities indirectly and interferes with the viral genome’s methylation and PTGS [74]. Additionally, C4 protein accessorizes Rep in the suppression of TGS via downregulation of MET1 and interaction with AGO4 [61,75]. The AC5/C5 ORF, located downstream of AC3/C3 in the complementary sense of DNA overlapping a region of the CP sequence, has been recently described as a potent suppressor of RNA silencing [76]. Mungbean yellow mosaic India virus (MYMIV) AC5 affects dsRNA production by suppressing sense ssRNA-induced gene silencing and interferes with TGS by inhibiting the expression of a CHH cytosine methyltransferase in *N. benthamiana* [76].

AV2 and V2 also suppress antiviral RNA silencing in the amplification phase and interfere with host methylation activities, downstream events of TGS [77,78,79]. TYLCV V2 interacts with and inhibits the suppressor of gene silencing 3 (SGS3), the cofactor of RDR6; thereby, affecting vsiRNA amplification [80]. V2 may also sequester the RDR6/SGS3 intermediate/substrate dsRNA with 5’ overhang ends from SGS3 association, further interfering with vsiRNA amplification [77,80]. Likewise, V2 from the curtovirus beet curly top virus (BCTV) has been recently shown to suppress post-transcriptional gene silencing (PTGS) by impairing the RDR6/SGS3 pathway [77,78]. CLCuMV V2 interacts with long dsRNA to prevent DCL processing, whereas tomato yellow leaf curl China virus (TYLCCV) V2 interacts with siRNAs to prevent AGO incorporation [70,81]. V2 also suppresses TGS by interacting with histone deacetylase 6 (NbHDA6) and interfering with the recruitment of MET1 by HDA6 resulting in decreased methylation of the viral genome and consequent increase in TYLCV pathogenicity [79]. Mulberry crinkle leaf virus (MCLV), a newly characterized geminivirus species, encodes a novel viral protein V3, which has been shown to suppress antiviral RNA silencing by a yet unknown mechanism [82]. CabLCV BVI (NSP) induces asymmetric leaves 2 (AS2) expression that activates DCP2 decapping activity and, in turn, accelerates mRNA turnover rate and inhibits siRNA accumulation [83].

Geminiviruses are often associated with alpha- and betasatellites, which encode efficient suppressors of viral genome methylation in infected plants and PTGS [84,85]. The betasatellite genome-encoded βC1 protein functions as an efficient VSR via different mechanisms [22]. TYLCCNV βC1 has been shown to inhibit *S*-adenosyl-L-homocysteine hydrolase (SAHH) activity, a methyl cycle enzyme required for TGS, interfering with the host methylation–mediated virus defense pathway [85]. Furthermore, βC1 from different betasatellites affects vsiRNA amplification by inducing the calmodulin-like protein (CaM), a negative regulator of RDR6 expression [86,87]. This scenario demonstrates that geminiviruses are very efficient in suppressing all steps in RNA-silencing-mediated antiviral mechanisms.

## 5. Does PAMP-Triggered Immunity Operate against Geminiviruses? What about Effector-Triggered Immunity?

**Figure 3:**
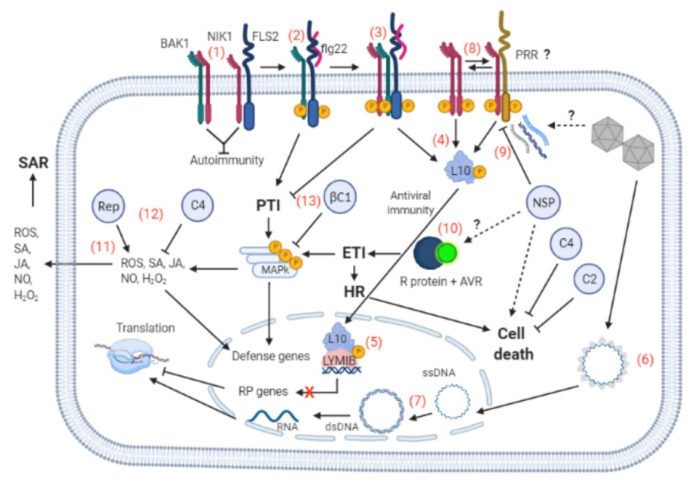
Antiviral innate immunity, interactions with viral suppressors and bacterial PTI (1). Under normal, resting conditions, NSP-interacting kinase 1 (NIK1) is bound to the flagellin receptor FLS2 (Flagellin-sensitive 2) and coreceptor BAK1 (Brassinosteroid insensitive 1-associated receptor kinase 1) to prevent autoimmunity. (2) Upon bacterial infection, the bacterial PAMP (pathogen-associated molecular pattern) flg22 is sensed by FLS2, inducing its oligomerization with BAK1, which results in phosphorylation-mediated activation of the immune complex to initiate PAMP-triggered immunity (PTI), activating the mitogen-activated protein kinase (MAPK) cascade and inducing defense genes. (3) Activated BAK1 also phosphorylates NIK1 at Thr-474, which in turn inhibits further PTI (4) and activates the NIK1-mediated antiviral signaling pathway, mediating ribosomal protein 10 (RPL10) phosphorylation. (5) Phosphorylated RPL10 is translocated to the nucleus, where it interacts with L10-interacting Myb domain-containing protein (LIMYB) to repress protein ribosomal (RB) genes and translational factors, suppressing global translation. (6) In infected cells, the geminivirus particles are disassembled in the cytoplasm and the CP-viral ssDNA complex is directed to the nucleus where (7) the viral ssDNA is converted into dsDNA for replication and transcription of the viral genome. (8) Begomoviruses-derived nucleic acids also function as viral PAMPs inducing NIK1 dimerization with itself or an unidentified viral PAMP recognition receptor (PRR) to transduce the antiviral signal that culminates in translational suppression. Then, viral mRNA is not efficiently translated impairing infection. (9) The begomovirus nuclear shuttling protein (NSP) counters the activation of this defense pathway by binding to the NIK1 kinase domain and, hence, prevents phosphorylation and transduction of the antiviral signal that otherwise would impair infection. (10) In resistant genotypes, NSP may also function as an avirulence (AVR) factor to activate effector-triggered immunity (ETI) via resistance (R) protein recognition, inducing hypersensitive response (HR) and cell death. (11) The PTI and ETI products (ROS, SA, JA, NO, H_2_O_2_) can induce defense genes and systemic acquired resistance (SAR). Geminivirus suppressors of innate immunity include (12) C4 that inhibits PTI by interacting and inhibiting FLS2 and along with Rep inhibits SA- and ROS-dependent signaling; C4 and C2 inhibit HR and cell death. (13) Furthermore, the betasatellite βC1 inhibits the MAP kinase cascade. The question marks indicate either events not well clarified or unknown. See Figure 1 for the designations of the viral proteins. The figure was created with BioRender.com.

In plant–virus interactions, the host immune system often recognizes viral components or effectors to activate defenses [2]. The plant antiviral innate defense consists of a two-level perception system represented by the cell surface receptor, PRR, and the intracellular immune receptors, the resistance (R) proteins [6]. PRRs are represented by two classes of transmembrane receptors, the receptor-like kinases (RLKs) and receptor-like proteins (RLPs). These PRRs recognize PAMPs exclusively expressed by pathogens, or endogenous danger signals released by host plants during infection, designated damage-associated molecular patterns (DAMPs) [88]. Activation of RLKs and RLPs often requires a coreceptor to form an active immune complex [89]. Mechanistically, PAMPs/DAMPs function as ligands that induce dimerization/oligomerization of single-pass transmembrane receptor PRRs with RLK coreceptors to initiate signaling via phosphorylation-induced activation of the immune complexes [88]. Downstream events of PTI activation include ROS accumulation, followed by MAP kinase cascade activation, induction of PTI-associated defense genes, ethylene and salicylic acid synthesis, and callose deposition [90] (Figure 3).

Although geminiviral PAMPs and their cognate PRRs have not been identified, several lines of evidence indicate that PTI is part of the host defense arsenal against geminivirus infection. First, the C4 protein from TYLCV has been shown to associate with a classical PRR, the bacterial flagellin receptor FLS2 (Flagellin-sensitive 2), and inhibits early PTI responses [14] (Figure 3). TYLCV C4 also interacts with NSP-interacting kinase 1 (NIK1), an antiviral immune leucine-rich repeat receptor-like kinase (LRR-RLK) that protects plants against begomoviruses [15]. NSP from CabLCV associates with the almost universal coreceptor of PRRs, the Brassinosteroid insensitive 1-associated kinase 1 (BAK1) [91]. NSP interaction with the BAK1 kinase domain may prevent phosphorylation and activation of the coreceptor in the same fashion as it does with the receptor-like kinase NIK1 [92]. Second, geminiviral proteins also activate and suppress downstream immune events of PTI activation (Figure 3). Rep from different geminiviruses induces PTI-associated marker genes and SA-dependent defenses [93,94]. However, when co-expressed with TYLCV C4 protein, Rep redirects C4 to the chloroplasts, where it acts as a PTI suppressor by reducing SA- and ROS-dependent defense signals [93]. The C4 immune-suppressing function co-opts a protein trafficking route directed by protein myristoylation/de-myristoylation processing [95]. Upon geminivirus infection, a fraction of the membrane-bound *N*-myristoylated C4 protein is de-myristoylated and translocated to the chloroplast. Inside the chloroplast, non-myristoylated C4 interacts with the thylakoid membrane-bound plant calcium-sensing receptor (CAS) and hampers SA biosynthesis and mediated defenses [95]. Finally, the betasatellite βC1 protein from TYLCCV has been shown to interfere with PTI-induced MAPK activation and downstream responses by targeting mitogen-activated protein kinase kinase 2 (MKK2) in *Arabidopsis thaliana* and *N. benthamiana* (Figure 3) [96,97,98].

The second level of microbe perception by the host plant is mediated by intracellular immune receptors (R proteins), which recognize pathogen avirulent effectors to activate ETI [3,93,99]. ETI represents a more specific and robust line of host defense, often associated with programmed cell death, HR that restricts the pathogen to the site of infection [100]. Most, but not all, antiviral R proteins harbor a nucleotide-binding leucine-rich repeat (NLR) domain, reminiscent of nonviral intracellular R proteins, which are further classified into coiled-coil (CC)-NLR or Toll/interleukin 1 receptor-like (TIR)-NLR proteins [2,6,101]. The natural Ty-2 resistance locus to TYLCV encodes an NB-LRR protein, named TYNBS1, which might mediate ETI-like resistance. However, the geminiviral effector that would interact specifically with TYNBS1 to activate ETI remains to be determined. Therefore, the underlying mechanism for TYNBS1 activation to mediate resistance is still elusive [17].

Compelling evidence that plants deploy ETI against geminiviruses results from infectivity assays demonstrating that geminiviruses induce and suppress HR and ETI-like responses. In *Arabidopsis*, CabLCV infection induces HR- and senescence-related genes without developing a visible cell death phenotype [102]. More specifically, TYLCV, cotton leaf curl Kokhran virus (CLCuKoV) and ACMV Rep, bean dwarf mosaic virus (BDMV) and bean golden yellow mosaic virus (BGYMV) NSP, and CLCuKoV V2 have been shown to induce HR (Figure 3) [81,103,104]. In contrast, the C2 proteins from the papaya leaf curl virus (PaLCuV) and CLCuKoV have been shown to inhibit V2-mediated HR, and C2 from tomato leaf curl New Delhi virus (ToLCNDV) suppresses NSP-mediated HR [105,106]. Likewise, TYLCV infection reduces cell death in tomato plants, which is induced by the inactivation of heat shock protein 90 (HSP90) and suppressor of the G2 allele of skp1 (SGT1), via an unknown TYLCV-mediated cell death suppression mechanism [107]. In contrast, the underlying mechanism for the cell death-suppressing activity of C4 from tomato leaf curl Yunnan virus (TLCYnV) has been recently unraveled [16]. C4 interacts with hypersensitive induced reaction 1 (HIR1), promotes its degradation by impairing HIR1 self-oligomerization, and hence inhibits the HIR1-mediated HR, increasing virus pathogenicity. Although several lines of evidence indicate that both monopartite and bipartite begomoviruses induce and suppress HR, with few exceptions, the mechanisms underlying these viral activities are still far from understood.

## 6. Transmembrane Receptor-Mediated Antiviral Immunity via Translational Suppression

As obligate intracellular parasites, viruses interact extensively with the host cell functions to complete their life cycle. Independent of the repertoire of viral genome–encoded proteins, all viruses are dependent on the host protein synthesis machinery to translate viral messenger RNAs (mRNAs) [108,109,110]. Therefore, many host cell-intrinsic immune defenses target translation factors to inhibit protein synthesis in the infected cells [108,110]. Among the translational control-mediated immune defenses, plant cells employ an LRR-RLK to sense viruses at the cell surface and activate a defense pathway that culminates in suppressing host and viral mRNA translation [12,111,112,113]. This translational control in antiviral immunity is mediated by the LRR-RLK NIK1, which was first identified as a virulence target of the begomovirus-encoded NSP [92,114]. NIK1 belongs to the subfamily II of LRR-RLK, which is further subdivided into phylogenetically related subclades, including a NIK antiviral subclade (NIK1-NIK3) and a somatic embryogenesis receptor kinase (SERK1-5) subclade of coreceptors in innate immunity [115]. In *Arabidopsis*, this subfamily of RLKs encompasses14 proteins that harbor four complete LRRs (with 24 residues) and a fifth incomplete LRR (with 16 residues) arranged in a single continuous block within the N-terminal extracellular domain, a single-pass transmembrane segment, and a highly conserved serine/threonine kinase domain at the C-terminal cytosolic side [116]. As a common property of the LRR-RLK subfamily II members, they often serve as coreceptors of multiple signaling receptors [89]. Accordingly, BAK1/SERK3, the best-characterized member of this subfamily II, acts as a coreceptor for the Brassinosteroid insensitive 1 (BRI-1) receptor in developmental signaling and several different PRRs in innate immunity. In addition to structural conservation, NIK1 also shares other properties with members of the LRR-RLK subfamily II [117]. The phosphorylation of the threonine (Thr) residue position 474 constitutes a critical regulatory mechanism for NIK1 activation [113,118]. Likewise, the activation site of BAK1/SERK3, SERK4, and SERK1 lies in a conserved position with NIK1 Thr-474 within the activation loop of the kinases [117]. Like BAK1/SERK4, which is activated upon PAMP-induced oligomerization of PRRs, NIK1 has been recently shown to be activated by begomoviruses-derived nucleic acids that may act as viral PAMPs. NIK1 may serve as a coreceptor for a yet unknown viral PAMP-sensing PRR.

According to the current model for the NIK1-mediated antiviral signaling, at the onset of infection, *Begomovirus*-derived nucleic acids act as viral a PAMP to activate NIK1 via phosphorylation on Thr-474 (Figure 3) [12,118]. As a member of the LRR-RLK subfamily II, NIK1 may function as a coreceptor of a yet-to-be-identified immune receptor that may recognize the viral PAMPs for the assembly of the active immune complex. Alternatively, NIK1 may undergo viral PAMP-induced dimerization with itself or its paralog NIK2. This latter hypothesis is based on in vitro phosphorylation assays demonstrating that NIK1 undergoes dimerization and autophosphorylation [92]. Phosphorylation-induced activation of NIK1 mediates the phosphorylation of the downstream component ribosomal protein (RP) L10 on the Ser-104 residue, which in turn is redirected to the nucleus [119,120]. Activated NIK1 also undergoes sequential autophosphorylation at Thr-469 that antagonistically inhibits NIK1 activation providing a mechanism to fine-tune the extent of RPL10 phosphorylation [117,118]. In the nucleus, RPL10 interacts with the transcriptional repressor L10-interacting Myb domain-containing protein (LIMYB) to fully repress the expression of ribosomal protein genes and translational regulatory factors [113]. Prolonged repression of translational-machinery-related genes leads to the suppression of global host translation. Begomoviral mRNAs cannot escape this translational regulatory mechanism of plant cells; they are not translated efficiently, impairing infection. NSP from begomoviruses counters this activation mechanism by binding to the NIK1 kinase domain and preventing phosphorylation at Thr-474, thereby enhancing *Begomovirus* pathogenicity [92,117]. In infected cells, RPL10 is trapped into the cytosol and is not translocated to the nucleus to mount the defense against begomoviruses. NIK1 overexpression titrates the viral suppressor NSP and restores the RPL10 nuclear localization upon NIK1 activation [118].

The NSP binding site on NIK1 was mapped to an 80-amino acid stretch within the activation loop, overlapping the essential Thr-474 for activation [92]. Therefore, NSP binding on the NIK1 kinase domain may cause stereochemical constraint on NIK1 phosphorylation at Thr-474, suggesting that NSP inhibition is upstream to NIK1 phosphorylation [120]. Consistent with this observation, the replacement of Thr-474 with the phosphomimetic aspartate residue creates a constitutively activated NIK1 mutant, NIK1-T474D, which is barely inhibited by the viral suppressor NSP [117]. Furthermore, expression of the gain-of-function mutant NIK1-T474D in *Arabidopsis* enhances resistance to CabLCV, and, in tomato plants, increases resistance to tomato yellow spot virus (ToYSV) and tomato severe rugose virus (ToSRV) [111,113]. This enhanced resistance phenotype has been associated with repression of translation-related regulatory genes, reduced loading of coat protein viral mRNA in actively translating polysomes, lower infection rate, and reduced viral DNA accumulation in systemic leaves [111]. However, a pitfall in this engineered defense strategy may be the side effects on development from impairing protein synthesis in transgenic crops under field growth conditions. Expression of NIK1-T474D in *Arabidopsis* causes stunted growth, although, in tomato plants, the transgenic lines are phenotypically indistinguishable from the untransformed lines under greenhouse standard conditions [111,113]. This difference in developmental performance between these plant species may be due to their differential intrinsic capacity to withstand the deleterious effect of translational inhibition under environmentally controlled conditions, which may not be sustained under field conditions.

Recent studies have shown that NIK1 not only plays an essential role in the translational control of antiviral immunity but regulates bacterial PTI negatively [121]. NIK1 interacts with the bacterial PAMP flagellin receptor, FLS2, and its coreceptor BAK1 to prevent autoimmunity under normal, non-infected conditions (Figure 3). However, during *Pseudomonas* infection, the bacterial PAMP flagellin induces FLS2 and BAK1 oligomerization and transphosphorylation, forming an active immune complex to initiate PTI signaling. The active FLS2–BAK1 immune complex, in turn, phosphorylates NIK1 on Thr-474, strengthening the NIK1 interaction with FLS2–BAK1 and concurrently activating the NIK1-mediated antiviral signaling. Significantly, bacterial flagellin (PAMP)-induced phosphorylation of NIK1 requires both FLS2 PRR and BAK1 coreceptor, indicating that NIK1 acts downstream of receptor signaling [121]. These results may implicate NIK1 as a coreceptor in receptor-mediated antiviral signaling that senses viral PAMPs via yet-unknown viral PRRs [12]. Furthermore, they demonstrate that a bacterial PAMP can induce NIK1 activation via the immune complex FLS2–BAK1, thereby allowing bacteria to activate antiviral immunity prior to virus infection. However, the finding that NIK1 suppresses antibacterial immunity further complicates the attempts to target NIK1-mediated antiviral signaling for engineered resistance against begomoviruses.

## 7. Conclusions

In RNA-silencing-mediated antiviral immunity, two mechanisms are fundamental to protect plants against invading nucleic acids from viruses, PTGS and TGS. PTGS is used as a defense mechanism against RNA and DNA viruses, whereas TGS targets virus DNA. PTGS acts at the post-transcriptional level by directing mRNA targets to degradation or translational suppression. In contrast, TGS represses transcription of target loci by controlling the chromatin methylation status and heterochromatin formation. Geminiviruses have evolved different suppressing strategies of host RNA-silencing-derived antiviral defenses. The geminiviral suppressors of RNA silencing can interfere with PTGS, TGS, and downstream events of TGS. They also act by activating or inducing the expression of endogenous suppressors. The immune-suppressing activities of geminiviruses may account for their broad host range and compromise the success of siRNA-based strategies for engineering host resistance.

The interactions of the plant’s innate immune system with geminiviruses are far less understood. Nevertheless, growing evidence has demonstrated that the classical plant PTI limits geminivirus infection similarly to nonviral pathogens. Geminiviral proteins have been shown to target PTI components to both induce and suppress PTI-like responses. Furthermore, begomoviruses-derived nucleic acids act as viral PAMPs and activate the transmembrane receptor NIK1, which shares with PTI coreceptors conserved regulatory mechanisms for activation. However, geminiviral PAMPs and their cognate PRRs have yet to be identified, and hence, the mechanism of geminiviral PTI activation remains unknown.

Likewise, plants may deploy ETI as part of the defense arsenal to combat geminiviruses. Accordingly, geminivirus infection induces ETI-like responses, including cell death, HR, ROS, SA accumulation in resistant genotypes, and at least one isolated resistant gene to TYLCV encodes an NLR protein, although the cognate geminiviral effector (Avr gene) is missing. The current studies designed to uncover geminiviral PTI- and ETI-suppressing functions are limited as they mostly target conserved features between the antiviral and anti-nonviral pathogen immunity. The discovery of geminiviral PAMPs and their cognate PRRs, intracellular R proteins, and the matching geminiviral effectors will potentially shed light on the mechanism of antiviral PTI and ETI activation uncovering specific and conserved features and their specific roles in resistance against viruses.

## Abbreviations

ACMV	African cassava mosaic virus
ADKs	Adenosine kinases
AGO	Argonaute
AS2	Asymmetric leaves 2
Avr	Avirulence
BAK1	Brassinosteroid insensitive 1-associated receptor kinase 1
BAM1	Barely any meristem 1
BCTV	Beet curly top virus
BDMV	Bean dwarf mosaic virus
BGYMV	Bean golden yellow mosaic virus
BRI-1	Brassinosteroid insensitive 1
BSCTV	Beet severe curly top virus
CabLCV	Cabbage leaf curl virus
CAS	Calcium-sensing receptor
CC-NLR	Coiled-coil nucleotide-binding leucine-rich repeat
CLCuKoV	Cotton leaf curl Kokhran virus
CLCuMuV	Cotton leaf curl Multan virus
CP	Coat protein
CTM3	Chromomethylase 3
DAMPs	Damage-associated molecular patterns
DCLs	Dicers
dsDNA	Double-stranded DNA
DRM2	DNA (cytosine-5)-methyltransferase 2
dsRNA	Double-stranded RNA
ETI	Effector-triggered immunity
FLS2	Flagellin-sensitive 2
H3K9	Histone H3 lysine 9
HEN1	Hua enhancer 1
HIR1	Hypersensitive induced reaction 1
HR	Hypersensitive response
HSP90	Heat shock protein 90
JA	Jasmonic acid
Kyp2/suvh4	suvh4/kryptonite2
LIR	Long intergenic region
LRR-RLK	Leucine-rich repeat receptor-like kinase
lncRNAs	Long non-coding RNAs
MAPK	Mitogen-activated protein kinase
MET1	Methyltransferase 1
miRNA	MicroRNA
MKK2	Mitogen-activated protein kinase kinase 2
MP	Movement protein
mRNA	Messenger RNA
MYMIV	Mungbean yellow mosaic India
MYMV	Mungbean yellow mosaic virus-*Vigna*
NbHDA6	*Nicotiana benthamiana* histone deacetylase 6
NB-LRR	Nucleotide-binding site-leucine-rich repeat
NIK1	NSP-interacting kinase1
NLR	Nucleotide-binding leucine-rich repeat
NSP	Nuclear shuttling protein
nt	Nucleotides
ORF	Open reading frame
Ori	Origin of replication
PaLCuV	Papaya leaf curl virus
PAMP	Pathogen-associated molecular pattern
pol	Polymerase
PRR	Pattern recognition receptor
PTGS	Post-transcriptional gene silencing
PTI	PAMP-triggered immunity
R	Resistance
RdDM	RNA-directed DNA methylation
RdRP or RDR	RNA-dependent RNA polymerase
REn	Replication enhancer protein
Rep	Replication initiator protein
rgs-CaM	Regulator of G-protein signaling calmodulin-like
RISC	RNA-induced silencing complex
RITS	RNA-induced transcriptional silencing complex
RLK	Receptor-like kinase
RNAi	RNA interference
ROS	Reactive oxygen species
RP	Ribosomal protein
SA	Salicylic acid
SAHH	*S*-adenosyl-L-homocysteine hydrolase
SAM	*S*-adenosyl methionine
SAMDC1	*S*-adenosyl-methionine decarboxylase 1
SAR	Systemic acquired resistance
SERK1-5	Somatic embryogenesis receptor kinase 1–5
SGS3	Suppressor gene silencing 3
SGT1	Suppressor of the G2 allele of skp1
SIR	Short intergenic region
siRNA	Small interfering RNA
ssDNA	Single-stranded DNA
ssRNA	Single-stranded RNA
TGMV	Tomato golden mosaic virus
TGS	Transcriptional gene silencing
Thr	Threonine
TIR-NLR	Toll/interleukin 1 receptor-like nucleotide-binding leucine-rich repeat
TLCYnV	Tomato leaf curl Yunnan virus
ToLCNDV	Tomato leaf curl New Delhi virus
ToSRV	Tomato severe rugose virus
ToYSV	Tomato yellow spot virus
TrAP	Transcriptional activator protein
TYLCCV	Tomato yellow leaf curl China virus
TYLCSV	Tomato yellow leaf curl Sardinia virus
TYLCV	Tomato yellow leaf curl virus
UTR	Untranslated region
vsiRNAs	Virus-derived short interfering RNAs
VSRs	Viral suppressors of RNA silencing
WDV	Wheat dwarf virus
WEL1	Werner exonuclease-like 1
